# Post-Mortem Appearances of the Body of Andrew J. Perteet (Colored), Executed at Joliet, Ill., Dec. 19, ’73

**Published:** 1874-03-15

**Authors:** Ben. Miller

**Affiliations:** Sanitary Superintendent, Chicago Board of Health


### POST-MORTEM APPEARANCES OF THE BODY OF ANDREW J. PERTEET (COLORED), EXECUTED AT JOLIET, ILL., DEC. 19TH, A.D., 1873.

REPORTED BY DR. BEN. MILLER, SANITARY SUPERINTENDENT, CHICAGO BOARD OF HEALTH.

THE crime for which this man was executed was that of uxoricide, committed some two years before. The act was done with a razor, the incision beginning at a point about an inch below the angle of the left jaw, partly severing the sterno-mastoid muscle, extending to the right and slightly downward in its course, severing the trachia and the carotid vessels, and pneumogastric nerve of the right side, and chipping off a portion of the transverse process of one the cervical vertebræ.

The gallows on which he expiated his crime was the ordinary upright post cross-beam, with a rope passing through pulleys, and attached to a heavy weight, which had a fall of eight feet. This was held by a lever. After being pinioned, the noose was placed about his neck, and the lever drawn. The falling of the weight, eight feet, threw his body into the air to a corresponding height, when it fell, with a sudden thud, returning about four feet.

The prisoner was a large, muscular man, with a neck measuring nineteen inches, and weighed two hundred and fifty pounds. I placed my finger on the pulse immediately after he dropped. At that time, no heart-beat was perceptible; but a spasm of the heart could be detected, which, after two or three attempts, began to pulsate, and during the first minute beat 48 strokes; the volume of the pulse was full and bounding, but the beats irregular; at the end of two minutes, still irregular, but weak, and beating 100 per minute; at three minutes, 120 beats per minute; at three and a half, 130, and very feeble; then it began to beat slower, with slightly increased force; and, at four minutes, had dropped to 60 beats per minute, and very irregular; at five minutes it had fallen to 52, the beats during the last half of the minute being scarcely perceptible; and then stopped; after ten or twelve seconds the heart made several feeble attempts to contract, and then stopped. The body hung thirty minutes, and was then cut down.

The countenance was natural; mouth firmly set, and the pupils of the eyes dilated. The first incision was made just forty minutes after the drop fell: the tissues warm; and the muscular fibers, upon irritation, would contract slightly.

The chest was first examined: lungs healthy; on right side, old pleuritic adhesions; the substance of the lungs was enormously engorged with blood, so much so that I can readily see how apoplexy of the lung might occur from the pressure.

The heart was healthy; on right side, a soft clot was present; the left side empty. The stomach contained about a pint of partly digested material, of the consistency of tomato soup, consisting of bread, eggs, and water. He had eaten no dinner; ate breakfast at eight A.M., and drank a glass of water just before the drop fell. The mucous membrane was covered with gastric fluid, and presented a beautiful rose-colored appearance. The villi seemed, to the naked eye, to be congested and erect, but perfectly healthy. The kidneys, spleen, and liver were healthy. The bladder was about one-third full of urine

The head was opened, and the membranes covering the brain exposed. They were found almost bloodless; but on cutting them, a quantity of serum exuded. The brain-substance proper presented the same appearance; and on cutting it, a large quantity of serum exuded. The brain weighed fifty-six and one-half ounces.

The neck was carefully examined, but was found not to be broken. The noose had slipped so that when removed from the neck it measured only thirteen and a half inches, being a compression of five and a half inches. Death was evidently caused by nervous shock, as he died very quickly and easily.

